# Supplementary material for: Enzymes and cellular interplay required for flux of fixed nitrogen to ureides in bean nodules
Source: Nat Commun. 2022 Sep 10;13:5331. doi: 10.1038/s41467-022-33005-5 (PMC9464200; doi:10.1038/s41467-022-33005-5)
Supplement: Supplementary file 1 — Supplementary Information [file 41467_2022_33005_MOESM1_ESM.pdf]

Enzymes and cellular interplay required for flux of fixed nitrogen to ureides in bean nodules

Luisa Voß, Katharina J. Heinemann, Marco Herde,

Nieves Medina-Escobar, Claus-Peter Witte

SUPPLEMENTARY INFORMATION

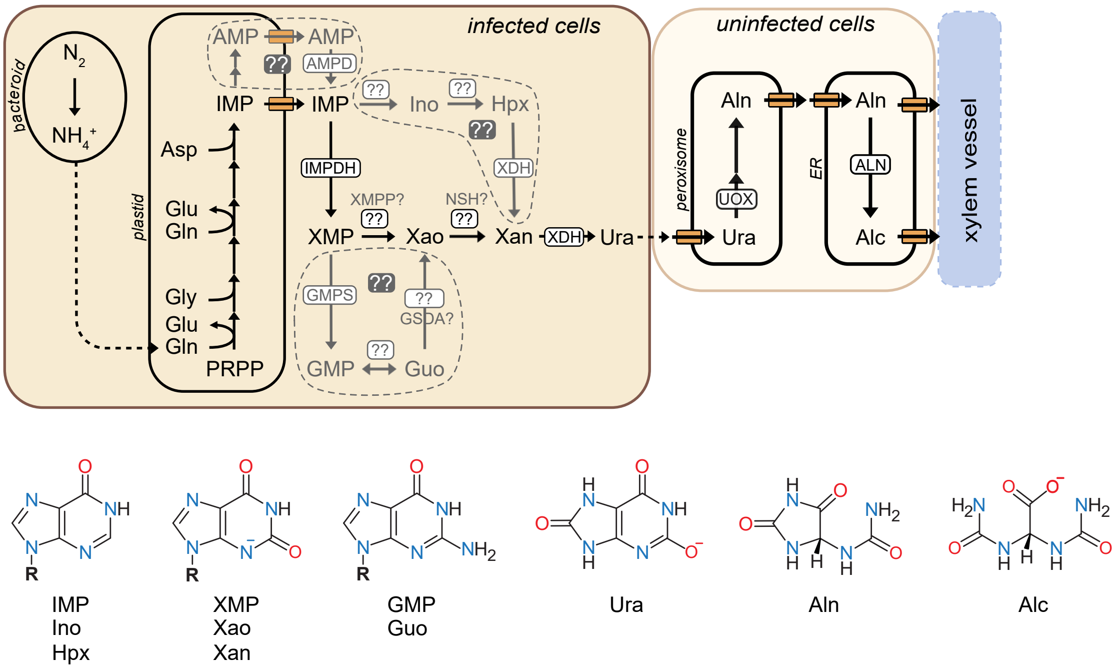

Supplementary Fig. 1 Current model of ureide biosynthesis in nodules and open questions.

The current model is in several aspects not based on ample experimental evidence obtained in vivo, but on in vitro data and on plausibility assumptions. Little support for pathways leading over AMP and GMP to ureides has been obtained in early metabolic labelling studies using cell-free extracts of cowpea[11,22]. These are grayed out and labelled '??' because their possible involvement has never been revisited but evidence available up to date is not sufficient to either include or exclude them. The pathway from IMP via hypoxanthine (Hpx) to xanthine (Xan) is also grayed out, because there is some evidence that it is not involved (see text). Although it is unknown which enzymes catalyze the reactions from XMP to xanthine (Xan) in vivo, it is assumed that the reactions take place in the infected cells. It is clear that the UOX reaction occurs in uninfected cells, thus some metabolite upstream of uric acid (Ura) is channeled from infected to uninfected cells – usually this is assumed to be Ura but sometimes also Xan since the cellular localization of XDH is controversial (here it is drawn in the infected cell). The ALN reaction downstream of UOX likely occurs in the ER of uninfected cells. It is known that not Ura but rather allantoin (Aln) and / or allantoate (Alc) are transported from the infection zone to the vascular tissue. Although these transport aspects are not shown in this model, it is known that ureide permeases (UPS, not shown) contribute to maintain Aln and Alc in the symplast so that they can reach the xylem vessels with high efficiency. Figure created with Affinity Designer by Serif.

Metabolites: PRPP, phosphoribosyl pyrophosphate; IMP, inosine monophosphate; AMP, adenosine monophosphate; Ino, inosine; Hpx, hypoxanthine, XMP, xanthosine monophosphate; Xao, xanthosine; Xan, xanthine; GMP, guanosine monophosphate; Guo, guanosine; Ura, urate; Aln, allantoin; Alc, allantoate. Enzymes: AMPD, AMP deaminase; IMPDH, IMP dehydrogenase; GMPS, GMP synthetase; XMPP, XMP phosphatase; NSH, nucleoside hydrolase; GSDA, guanosine deaminase; XDH, xanthine dehydrogenase; UOX, urate oxidase; ALN, allantoinase. ER, endoplasmic reticulum.

'R' in the chemical formulas is either a proton for the nucleobases (Hpx, Xan) or ribose for the nucleosides (Ino, Xao, Guo) or ribose-5-phosphate for the nucleotides (IMP, XMP, GMP).

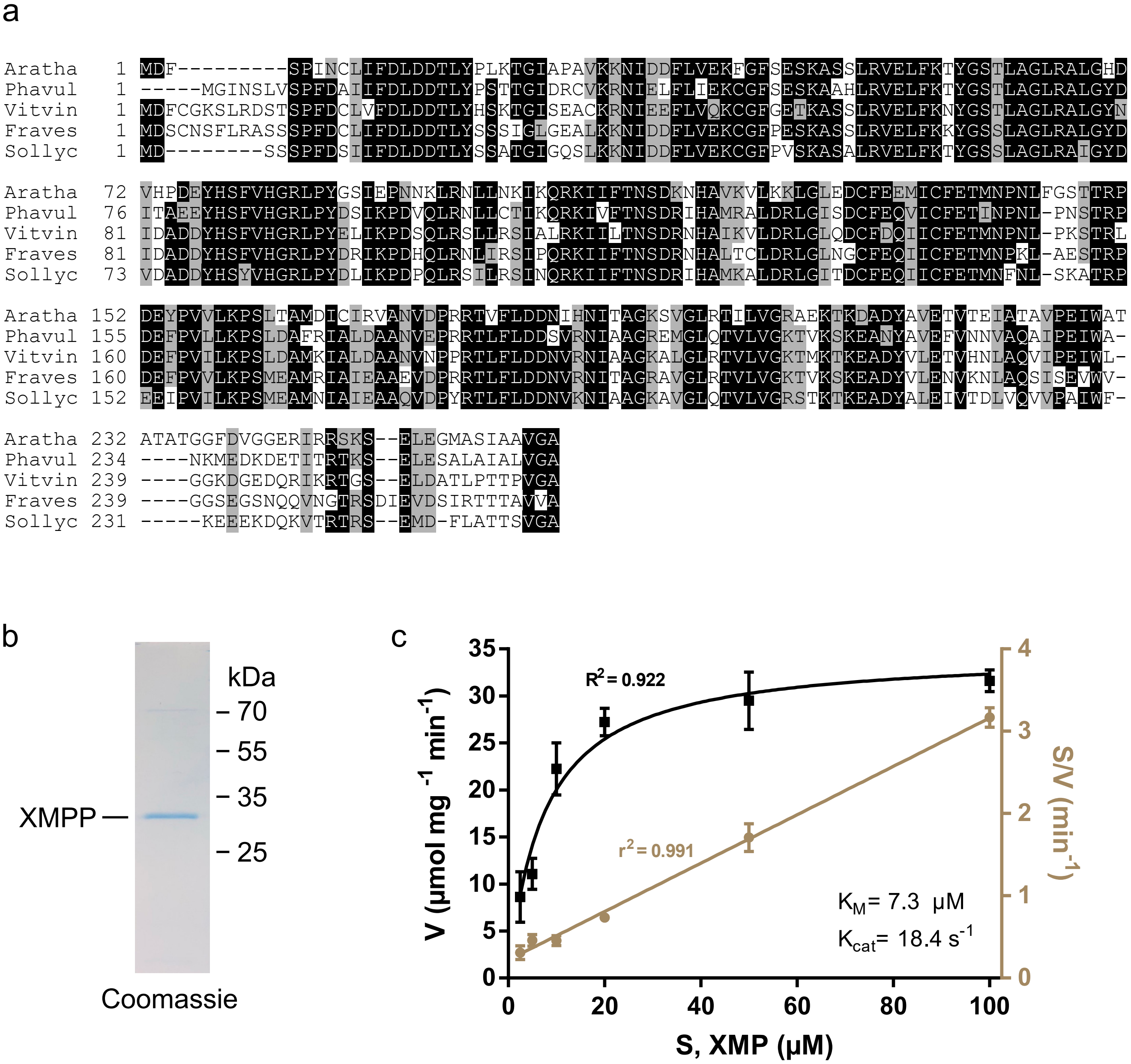

Supplementary Fig. 2 Multiple alignment of XMPP from five dicot species and biochemical characterization of XMPP from *Phaseolus vulgaris.*

a XMPP protein sequences from *Arabidopsis thaliana* (Aratha, At2g32150.1), *Phaseolus vulgaris* (common bean, Phavul, Phvul007G056000.1), *Solanum lycopersicum* (tomato, Sollyc, Solyc09g075890.3.1), *Fragaria vesca* (strawberry, Fraves, FvH4_4g15270.t1), and *Vitis vinifera* (grape vine, Vitvin, VIT_205s0020g03190.1) were gathered from the Phytozome V13 database. From the *P. vulgaris* sequence, 72 N-terminal amino acids have been removed because these are wrongly annotated according to the sequence consensus and the cDNA we have cloned. The sequences were aligned using MUSCLE at EBI and shaded with pyBoxshade. **b** Coomassie-stained SDS gel with C-terminal HAStrep-tagged XMPP, affinity purified after transient expression in *Nicotiana benthamiana*. **c** Determination of the kinetic constants with the data fitted according to Michaelis Menten (left axis) or Hanes (right axis). Error bars are SD, measure of the center is the mean value, *n* = 3 technical replicates. Experiment was performed twice with similar results.

According to results with the Arabidopsis enzyme[15], the kinetic constants of XMPP are not significantly different irrespective of whether the protein is tagged at the N- or C-terminus. Structural data revealed that the C-terminus is not involved in forming the active site and can also be truncated without loss of activity.

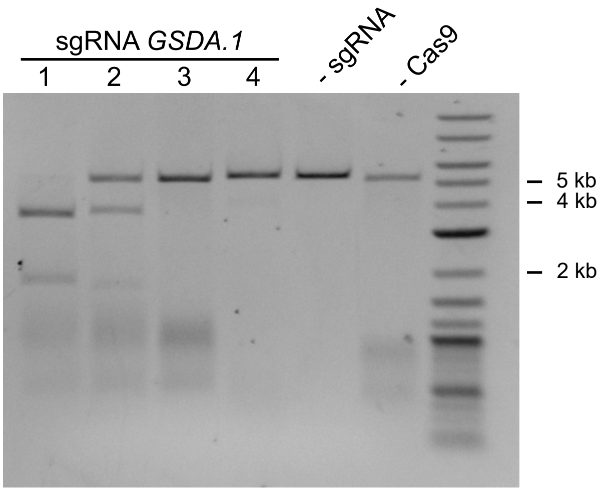

Supplementary Fig. 3 Cas9 in-vitro cleavage assay.

Cas9-mediated DNA cleavage of *GSDA.1*. Four different sgRNAs were tested, and cleavage of the template DNA fragment was assessed. Controls either lacked sgRNA or Cas9. The template was the genomic DNA sequence of *GSDA.1*, which was subcloned in pJET1.2 (ThermoFischer) and linearized with *Cla*I. Experiment was performed twice with similar results.

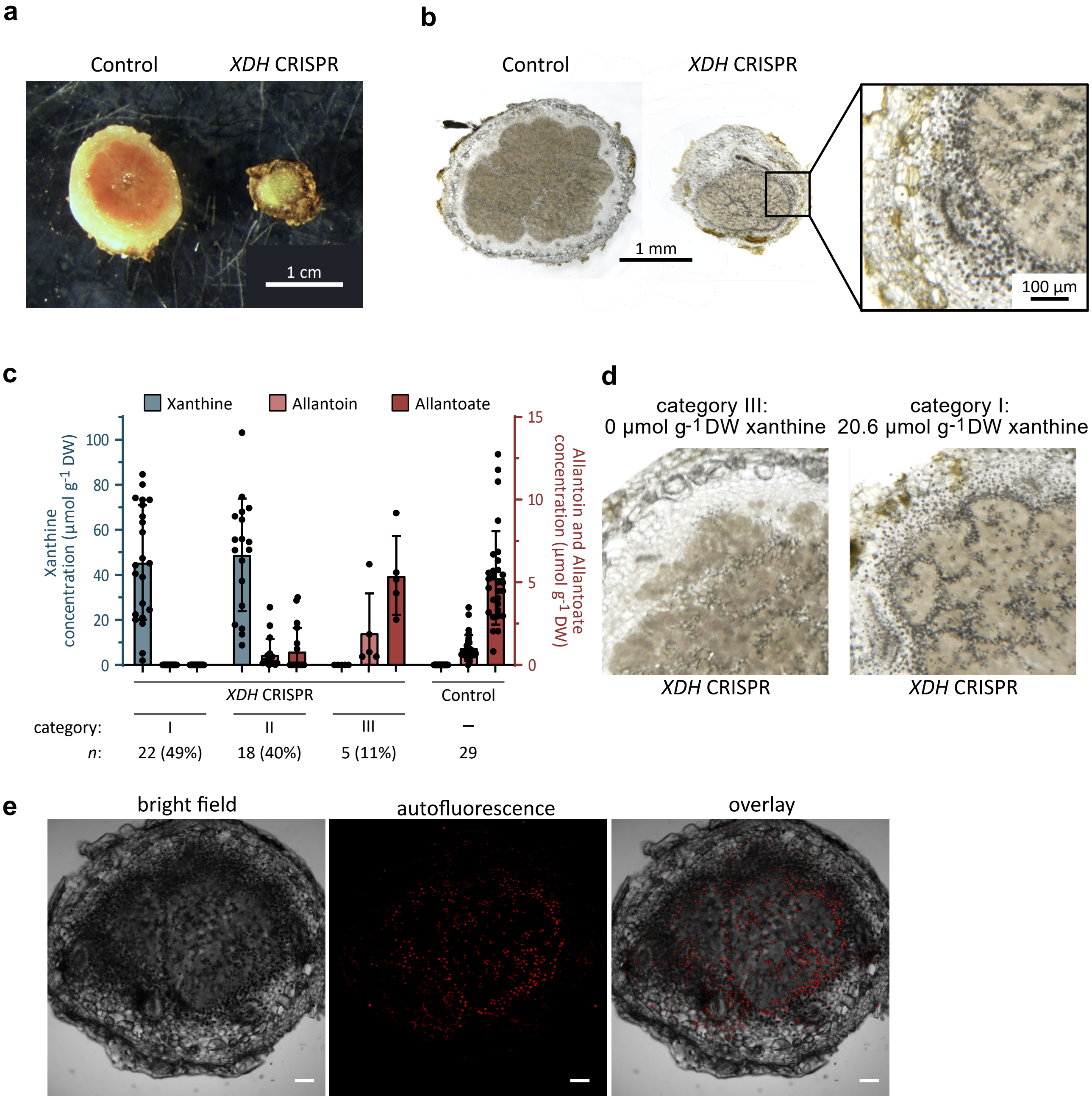

Supplementary Fig. 4 Characterization of *XDH* CRISPR mutant nodules in soybean.

a *XDH* CRISPR mutant nodules are smaller and lack leghemoglobin as has been reported recently[14]. **b** *XDH* CRISPR mutant nodules show black spots associated with the uninfected cells in or close to the infection zone. **c** 45 *XDH* CRISPR nodule pools of soybean, i.e. hairy roots with nodules transformed with the *XDH* CRISPR construct without testing the resulting edited genotypes, were analyzed for their xanthine, allantoin and allantoate content and grouped into categories: I, accumulating xanthine but not ureides – these were putative complete null mutants of *XDH*; II accumulating xanthine and some ureides – these were probably incomplete mutants; III, not accumulating xanthine but ureides – in these XDH activity was probably not abolished. Controls had been transformed with a vector lacking sgRNAs. Error bars are SD, measure of the center is the mean value, n = 22 for category I, n = 18 for category II, n = 5 for category III and n = 29 for control. A repeat (n) is a pool of nodules from a single transgenic root. **d** Only nodules accumulating xanthine had black dots as described in b. **e** The black dots show autofluorescence detected between 595 and 622 nm when excited with 452 nm laser light in the confocal microscope similar to the xanthine co-crystals described in the xanthine accumulating *xdh* background of Arabidopsis[43]. Scale bar, 100 µm. Images are representative of at least three nodules from independent transgenic roots. Experiment was performed twice with similar results.

This example shows that CRISPR system can be used to generate mutations in hairy roots and nodules of soybean, which has recently also been demonstrated by Nguyen et al. (2021)[14]. However, about 75% of the soybean genome is duplicated which makes mutational approaches more laborious than in common bean. In soybean there are for example two *XMPP* genes and five *GSDA* genes compared to one *XMPP* and three *GSDAs* in common bean.

a

```
Aratha   1 MDCGMENCNGGISNGD----VLGKH---EKLIIDTDPGIDDSMAILMAFQTPELEILGLTTVFGNVSTQDATRNALLLCE
Phavul   1 MASQVNSPNGAV------NGVLGNA---EKLIIDTDPGIDDSMAILMAFQSPDVEVLGLTSIFGNAMTEVSTRNALLLCE
Fraves   1 MGTLMTNSDTAVAIEGRSDGVLGCNLKRDKLIIDTDPGIDDTMAILMAFQTPELEILGLTTIFGNVTTEDATRNALLLCE
Sollyc   1 ---------MSICDGDLVDSNNSFAKQREKIIIDTDPGIDDSMTILMAFQTPEVEIIGLTTIFGNVTTKDATRNALLLCE
Vitvin   1 MECVMLSSHGGLCDASY-DVVSNSPVQPDKVIIDTDPGIDDSMAILMAFQTPELEILGLTTVFGNVTTKDATRNALLLCE

Aratha  74 IAGFPDVPVAEGSSEPLKGGIPRVADFVHGKNGLGDVSLPPPSRKKSEKSAAEFLDEKVEEYPGEVTILALGPLTNLALA
Phavul  72 IAGRENVPVAQGSSEPLKGGTPRIADFVHGEDGLGNTFLPLPKGKKIEKSACEFLVEKVSENPGEVSVLALGPLTNVALA
Fraves  81 IAGQPGLPVAEGSHEPLKGGRPRVADFIHGSDGLGNVFIPPPETKKIEKNAAEFLVDTVSQYPGEVSILALGPLTNLALA
Sollyc  72 AAGYPDVPVAEGSPEPLKGGEPRVADFVHGSDGLGNLFLPSPNSKKIDKSASEFLVEKVSEYPGEVSILALGPLTNLALA
Vitvin  80 IAGRPDVPVAEGSSGPLKGGEPRVADFIHGSDGLGNIFLPQPKAKKIEKNAAEFLVDKVSEYPGEVSILALGPLTNVALA

Aratha 154 IKRDSSFASKVKKIVILGGAFFSLGNVNPAAEANIYGDPEAADVVFTSGADITVVGINITTQLKLSDDDLLELGNCKGKH
Phavul 152 IKRDSSFASKVKRIVILGGSFFALGNVNPAAEANIYGDPEAADVVFTSGADIVVVGINITTQVQFTDADLIQLKESQGKY
Fraves 161 IKRDSSFVKKVKRVVVLGGAFFALGNVNPAAEANIYGDPEAADVVFTSGANITVVGINITTQVKFTDSDLLQLRQSKGKH
Sollyc 152 VKRDSTFASKVKRVVVLGGSFFALGNVNPAAEANIYGDPEAADVVFTSGANIDVVGINITTQVKLKDADLEELKQSKGKY
Vitvin 160 IKRDSSFASKVKKVVVLGGAFFALGNVNPAAEANIYGDPEAADVVFTSGANIVVVGINITTQIKFTDADLHQLRHSEGRY

Aratha 234 SKLISDMCKFYRDWHVKSDGVYGVYLHDPVSFVAVVRPDLFTYKKGVVRVETQGICVGHTLMDQGLKRWNGSNPWVGYSP
Phavul 232 APFLSDICKFYRDWHAKSDGVHGIFLHDPVSFVALVRPDLFTYQKGVVRVETQGICVGHTLLDQGLKNWNMSNPWTGYSP
Fraves 241 VQFITDTCQFYRDWHVKSDSVYGIFLHDPVSFVAVVRPDLFTYKKGVVRVETQGICVGHTLMDQGLKNWNSSNPWTGYSP
Sollyc 232 AKFVCDMCKFYRDWHVKSDGVYGIFLHDPVSFAALVWPELFTFKKGVVRVETQGICVGHTLMDQGLKKWNTSNPWTDYSP
Vitvin 240 AQFISDICKFYRDWHVKSDGVYGIFLHDPVSFAALVRPDLFTYKEGVVRVETQGICLGHTLMDQGLKIWNSSNPWTGYSP

Aratha 314 ISVAWTVDVEGVLEYVKAKLMKP
Phavul 312 VSVVWTVDVEGVVDYIKELVMKP
Fraves 321 VEVAWTVNVDGVLDYIKDRLMAS
Sollyc 312 VSVAWTVDVDEVLDYIKKTLMKP
Vitvin 320 VSVAWTVDVDGVLNYIKKLLMKL
```

b

```
Aratha   1 -MAIGDRKKIIIDTDPGIDDAMAIFVALNSPEVDVIGLTTIFGNVYTTLATRNALHLLEVAGRTDIPVAEGTHKTFLNDT
Phavul   1 MAADTEPKKIIIDTDPGIDDAMAIFVALQSPEIEVIGLTTIYGNVYTTLATRNALHLLEVAGRTDIPVAEGTHLTLTKGT
Sollyc   1 --MATEPKKIIIDTDPGIDDAMAIFVALESPEVEVIGLTTIYGNVYTTLATRNALHLLDIAGRTDIPVAEGSHVTITKGT
Fraves   1 -MAVTDPKKIIIDTDPGIDDAMAIFVALKSPEVEVIGLTTIYGNVYTTLATRNALHLLEFAGRTDIPVAEGSHVTITQGT
Vitvin   1 ---MAEPKKIIIDSDPGIDDAMAIFVALQSPEVDVIGLTTIYGNVYTTLATRNALHLLEIAGRTDIPVAEGSHVTITKGT

Aratha  80 KLRIADFVHGKDGLGNQNFPPPKGKPIEKSGPEFLVEQAKLCPGEITVVALGPLTNLALAVQLDPEFSKNVGQIVLLGGA
Phavul  81 KLRIADFVHGADGLGNQNFPPPKGKPIEESAASFLVRQAKLNPGKVTVVALGPLTNIALAIELDPEFVKNIGQIVILGGA
Sollyc  79 KLRIADFVHGTDGLGNQNFPAPNGKPIEQNAADFLVQQASLYPGKITVVALGPLTNIALAIQSDPDFVKNIGQIVVLGGA
Fraves  80 KLRIADFVHGTDGLGNQNFPPPKGKPIEQSAAAFLVEQANLHPGKVTLVALGPLTNIALAIQLDPAFAKNIGQIVLLGGA
Vitvin  78 KLRIADFVHGADGLGNQNFPPSAGKPIEQSAAAFLIEQAKLYPGKVTVVALGPLTNIALAIELDPGFSKNIGQIVLLGGA

Aratha 160 FAVNGNVNPASEANIFGDPEAADIVFTCGADIIAVGINVTHQVIMTADDKDKLASSKGKLAQYLCKILDVYYDYHLTAYE
Phavul 161 FAVNGNVNPAAEANIFGDPDAADVVFTSGADILALGINVTHQVILTSSDREILASSNGKFAQYLHKILDVYFSYHQESYN
Sollyc 159 FSVNGNVNPAAEANIFGDPDAADIVFTSGADVLAVGINVTHQVVLTDFDRDELAKSNGKYAKYLDKILGVYFGYHHEAYS
Fraves 160 FAVNGNVNPAAEANIFGDPDAADIVFTSGADVLAVGINVTHQVVLTDADRDKLAKSNGKFAQYLCKILDVYFSYHHDAYS
Vitvin 158 FAVNGNVNPAAEANIFGDPEAADIVFTSGADILAVGINVTHQVVLTDADREKLAQSNGKFAQYLCKILEVYFSYHRDAYN

Aratha 240 IKGVYLHDPATILAAFLPSLFTYTEGVARVQTSGITRGLTLLYNNLKRFEEANEWSDKPTVKVAVTVDAPAVVKLIMDRL
Phavul 241 TKGVYLHDPTVVLAAVDPSLVTCIEGIVRVQTSGITRGLTLLYNKQKRFGEIHEWSNKPTVKVAVTVDAPRVMKLVMDRL
Sollyc 239 TKGVYLHDPTALLAAVDPSLITYTEGVVRVQTNGITRGLTLFYNKQKRFGEVSEWSDKPSVKVAVTVDAPRVLKLVMERL
Fraves 240 TKGVYLHDPTTLLAAVNPSLLTYTEGAVRVQTSGITRGLTILYNKQKNFGEVTEWCDKPTVKVAVTVDAPTVLNLVMERL
Vitvin 238 TKGVYLHDPTTLLAAVNPSLITYTEGVVRVQTSGITRGLTLFYNKQKRFAEVTEWTDKPTVKVAVTVDAPAVVKLVMDRL

Aratha 320 MES
Phavul 321 LDS
Sollyc 319 INS
Fraves 320 MDS
Vitvin 318 IDS
```

Supplementary Fig. 5 Multiple alignments of NSH1 and NSH2 from five dicot species.

NSH1 and NSH2 protein sequences from *Arabidopsis thaliana* (Aratha), *Phaseolus vulgaris* (common bean, Phavul), *Solanum lycopersicum* (tomato, Sollyc) ,*Fragaria vesca* (strawberry, Fraves), and *Vitis vinifera* (grape vine, Vitvin) were gathered from the Phytozome V13 database, aligned using MUSCLE at EBI and shaded with pyBoxshade. Note that the C-termini are more conserved than the N-termini. **a** NSH1. **b** NSH2.

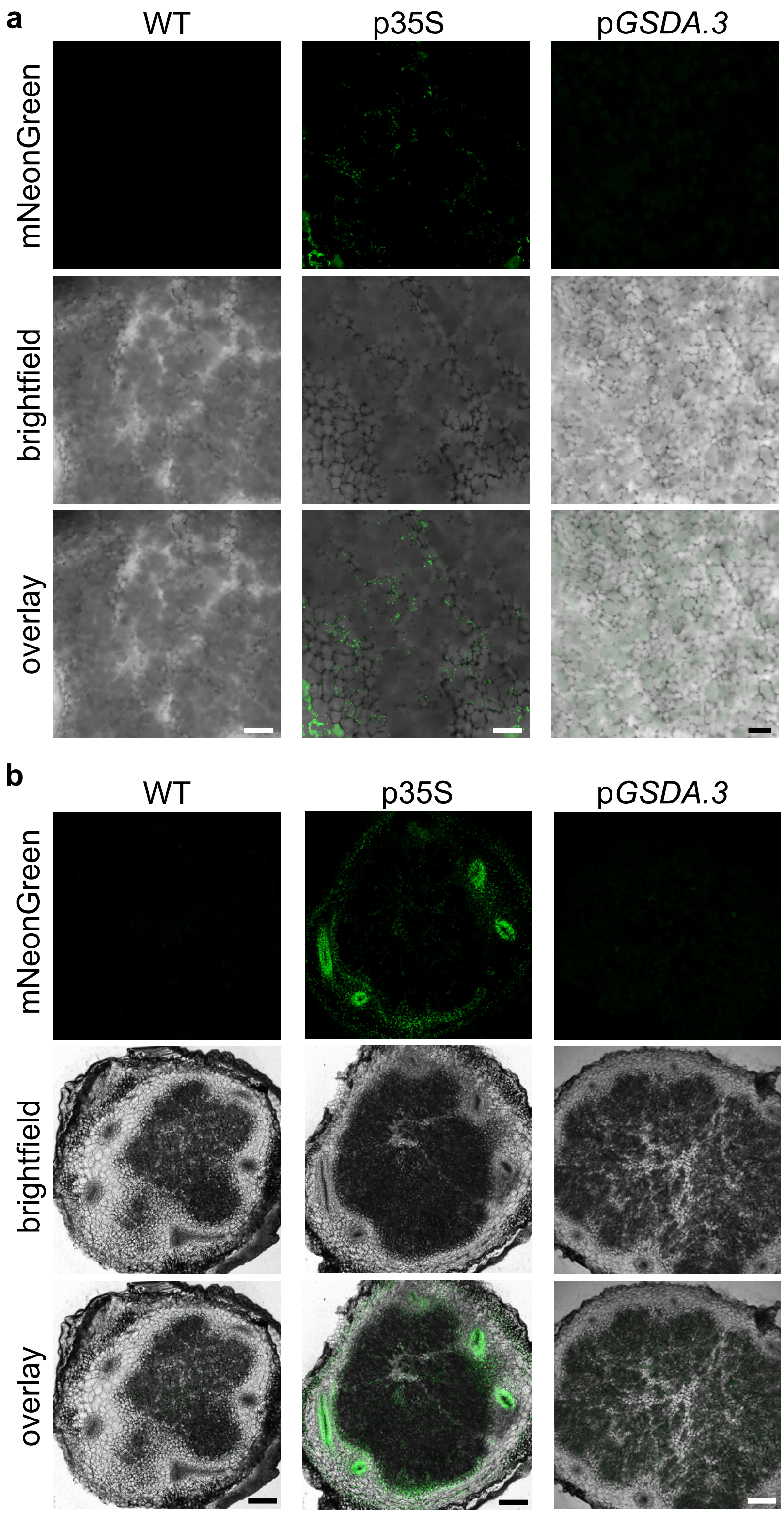

Supplementary Fig. 6 Cell-type specific activity of the 35S and *GSDA.3* promoters in the common bean nodule.

Confocal fluorescence microscopy images of cross sections from wild type (WT) nodules or nodules expressing the coding sequence of peroxisome-targeted mNeonGreen under the control of the Cauliflower Mosaic Virus 35S promoter or the *GSDA.3* promoter. **a** Focus on the central infected region, consisting of infected (dark grey) and uninfected (light grey) cells. Scale bar, 100 µm. **b** Whole nodules. Scale bar, 225 µm. From top to bottom, mNeonGreen channel, brightfield channel and overlay of both channels. Images are representative of at least three nodules from independent transgenic roots. The experiment was performed twice with similar results.

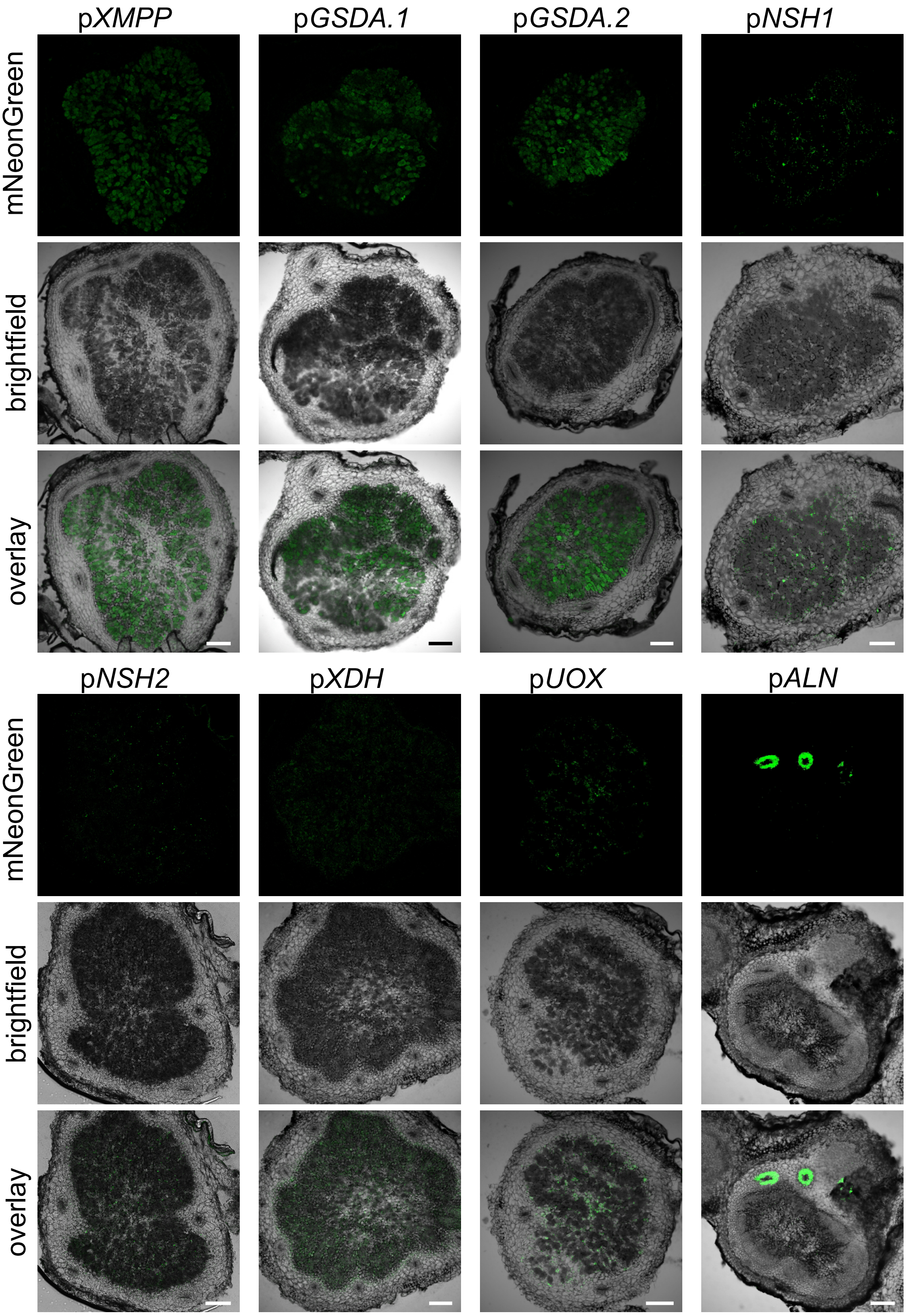

Supplementary Fig. 7 Whole nodule images of cell-type specific activity of ureide biosynthesis gene promoters in common bean nodules.

Confocal fluorescence microscopy images of cross sections from nodules expressing the coding sequence of peroxisome-targeted mNeonGreen under the control of native promoters of genes involved in ureide biosynthesis. Similar to Fig. 5 but the whole nodule is shown. From top to bottom, mNeonGreen channel, brightfield channel and overlay of both channels. Scale bars, 100 µm. In the brightfield images infected cells appear dark grey and uninfected cells light grey. Images are representative of at least three nodules from independent transgenic roots. The experiment was performed twice with similar results.

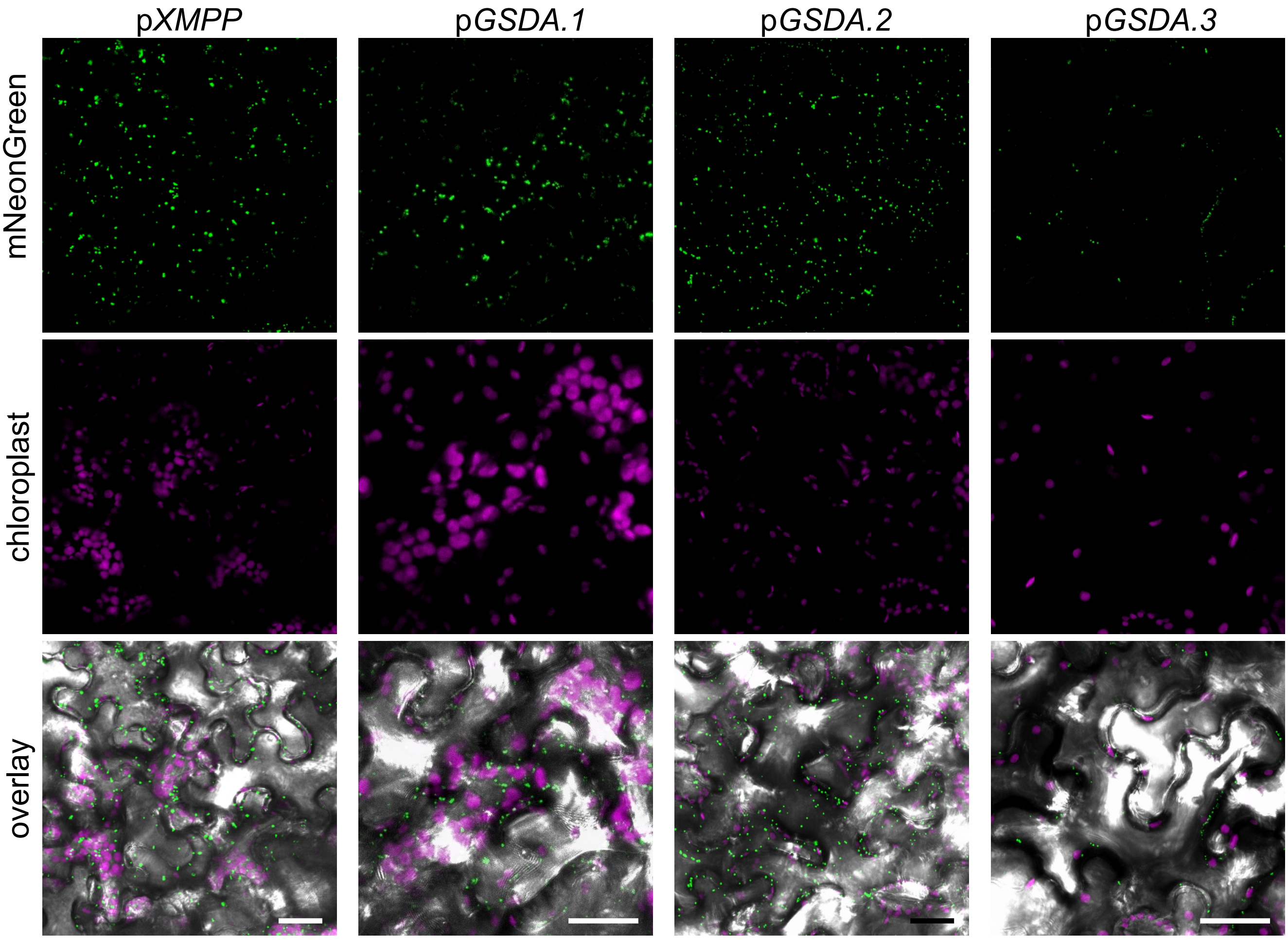

Supplementary Fig. 8 Subcellular localization of peroxisome-targeted mNeonGreen expressed from constructs with ureide biosynthesis gene promoters in leaves of *N. benthamiana*.

Confocal fluorescence microscopy images of the lower epidermis of *N. benthamiana* leaves expressing peroxisome-targeted mNeonGreen from constructs used in bean nodules to study cell-specific promoter activity. p*XMPP*, p*GSDA.1*, p*GSDA.2*, p*GSDA.3* indicate the promoters of the respective constructs. From top to bottom, mNeonGreen channel, chloroplast autofluorescence channel, overlay of both channels including brightfield images. Scale bars, 50 µm. The experiment was performed twice with similar results.

Because mNeonGreen was fused to a C-terminal peroxisome-targeting sequence 1 (SKL), a peroxisomal localization was expected. A punctuate pattern of mNeonGreen was obtained for all tested constructs consistent with peroxisomal localization, confirming the functionality of the constructs. By contrast, when the same constructs were expressed in bean nodules, resulting in activity in infected cells (Fig. 5 and Supplementary Fig. 7), a punctuate fluorescent pattern was not observed. This indicates, that mNeonGreen cannot be targeted to the peroxisomes in infected nodule cells. However, for constructs with the *XDH* promoter, a punctuate fluorescence signal was observed in infected cells. We speculate that these fluorescent spots do not represent peroxisomes in this case but may result from a spatial confinement of *XDH* promoter activity or translation activity possibly conferred by the 5'-leader sequence – all promoters were cloned with 5'-UTR sequences ending right upstream of the AUG start codon, i.e. the 5'-UTR sequences of the respective genes were present in all reporter constructs upstream of the mNeonGreen coding sequence.

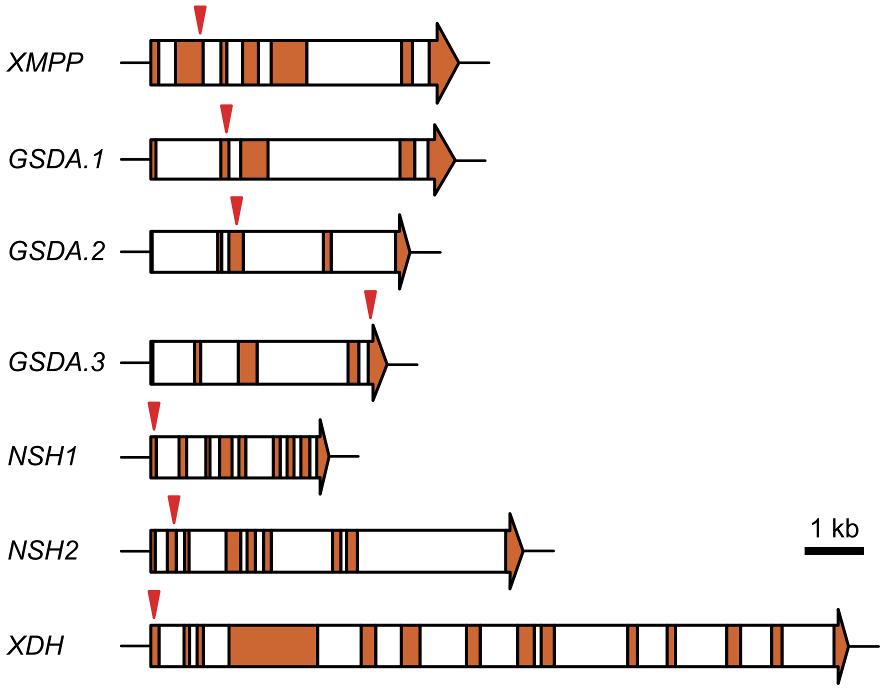

Supplementary Fig. 9 Phaseolus genes and sgRNA binding sites.

Schematic overview of the sgRNA binding sites (red rectangles) on coding sequences of *P. vulgaris* genes. Exon and intron structures within the coding region are displayed in orange and white, respectively.

Supplementary Table 1 List of oligonucleotides and synthetic DNA fragment.

Name	Sequence 5’ to 3’	Description
P 272	TAGGTCTCCAAACGAAGACAAAAACAAAAAAAAAAAGCACCGACTCG	CRISPR sgRNA production with tRNA processing machinery
P 274	TAGGTCTCCAAACGAAGACAAAAAC	CRISPR sgRNA production with tRNA processing machinery
P 293	CGGGTCTCAGGCAGAAGACTAATTGAACAAAGCACCAGTGG	CRISPR sgRNA production with tRNA processing machinery
P 294	CGGGTCTCAGGCAGAAGACTAATTG	CRISPR sgRNA production with tRNA processing machinery
P 626	TTGAAGACAAAATGGAGTCTGATGAGTCT	To amplify turbo-GFP
P 627	TTGAAGACAAAAGCTTATCATTCCTCACC	To amplify turbo-GFP
P 824	TTGAAGACAAGCTTTTAACTCTGGTTTCATT	To amplify on V69 CPMV3-UTR+35STerm for 3U-Ter module (pICH41276)
P 825	TTGAAGACAAAGCGGGTCACTGGATTTTG	To amplify on V69 CPMV3-UTR+35STerm for 3U-Ter module (pICH41276)
P1481	TGCCGGCGCGCCAAGCGGCCGCTTAATTAACCTAGGAAAAGCGATCGCGACGTCGAAGTGA TCCGTTT	Oligonucleotide for multiple cloning site in pY vectors, contains *Asc*I, *Not*I, *Pac*I, *Avr*II, *AsiS*I, *Aat*II, forward
P1482	AAACGGATCACTTCGACGTCGCGATCGCTTTTCCTAGGTTAATTAAGCGGCCGCTTGGCGC GCC	Oligonucleotide for multiple cloning site in pY vectors, contains *Asc*I, *Not*I, *Pac*I, *Avr*II, *AsiS*I, *Aat*II, reverse
P1485	TCCATGGACGACGCTACTTCTG	To amplify *GSDA.1* (Phvul.007G185600) from gDNA; template (in pJET1.2) for the Cas9 cleavage assay; forward
P1486	ACCCGGGTCAGTACAGAGTAAACTTCTCTTTTGTC	To amplify *GSDA.1* (Phvul.007G185600) from gDNA; template (in pJET1.2) for the Cas9 cleavage assay; reverse
P1491	TGGCGCGCCTGAAATCCACACAGCTATGC	To amplify *P. vulgaris XMPP* promoter, Phvul.007G056000, forward with *Asc*I site
P1492	ACTCGAGTGTAGGAGAAGAAGAGGAAGAAG	To amplify *P. vulgaris XMPP* promoter, Phvul.007G056000, reverse with *Xho*I site
P1497	TGGCGCGCCTGCTTAGGTTTTAGCGTGTTTAG	To amplify *P. vulgaris GSDA.1* promoter, Phvul.007G185600, forward with *Asc*I site
P1498	ACTCGAGGATGGTATTGGGATCTGAGG	To amplify *P. vulgaris GSDA.1* promoter, Phvul.007G185600, reverse with *Xho*I site
P1501	TGGCGCGCCTGAGACAAGGATTGTTATTGC	To amplify *P. vulgaris GSDA.2* promoter, Phvul.009G220800, forward with *Asc*I site
P1502	ACTCGAGTTTGTTGGTGTTATGAACTAACG	To amplify *P. vulgaris GSDA.2* promoter, Phvul.009G220800, reverse with *Xho*I site
P1505	TGGCGCGCCTAGGAACAAGCTAGAATGCTAC	To amplify *P. vulgaris GSDA.3* promoter, Phvul.003G124100, forward with *Asc*I site
P1506	TCTCGAGAGGAGCGTTCGATTAATGAAG	To amplify *P. vulgaris GSDA.3* promoter, Phvul.003G124100, reverse with *Xho*I site
P1509	TGGCGCGCCTCCATGTCATCCTTAGC	To amplify *P. vulgaris NSH1* promoter, Phvul.001G188700, forward with *Asc*I site
P1510	ACTCGAGGATCTGATCGGAGAAGAGCTAG	To amplify *P. vulgaris NSH1* promoter, Phvul.001G188700, reverse with *Xho*I site
P1513	TGGCGCGCCAGATTTTCATTTAGATGTGTTGAG	To amplify *P. vulgaris NSH2* promoter, Phvul.003G000600, forward with *Asc*I site
P1514	ACTCGAGATCTTGCAGAACTAATGACTTTACTTG	To amplify *P. vulgaris NSH2* promoter, Phvul.003G000600, reverse contains *Xho*I site
P1515	TGGCGCGCCCATGTTTCTTAATGTTTATCAC	To amplify *P. vulgaris XDH* promoter, Phvul.005G148000, forward with *Asc*I site
P1516	ACTCGAGGAAACGAACGGAACGG	To amplify *P. vulgaris XDH* promoter, Phvul.005G148000, reverse contains *Xho*I site
P1517	TGGCGCGCCGATTATAAAATTTCTCGATTCTCTTG	To amplify *P. vulgaris UOX* promoter, Phvul.007G234300, forward with *Asc*I site
P1518	ACTCGAGCTTTTCGGAATAAACACTGTTTAG	To amplify *P. vulgaris UOX* promoter, Phvul.007G234300, reverse contains *Xho*I site
P1521	TGGCGCGCCATGTTTCATAGCTAATAAGCATCG	To amplify *P. vulgaris ALN* promoter, Phvul.006G186700, forward with *Asc*I site
P1522	ACTCGAGTTCTCACCAATTCACCATTG	To amplify *P. vulgaris ALN* promoter, Phvul.006G186700, reverse contains *Xho*I site
P1541	TGTTTAAACTAAGAGAAAAGAGCGTTTATTAG	To amplify a fragment from pAGM4723 (corrected sequence - Addgene) binary plasmid to remove right border, contains *Pme*I site, forward
P1542	GTGGCCCATATGTCGTAAG	To amplify a fragment from pAGM4723 (corrected sequence - Addgene) binary plasmid to remove right border, contains *Nde*I site, reverse
P1543	AAACTCAGTGTTTGACAGGATATATTGGCGGGTAAACTTGTTCAATTGTTCAATTGTTCAA TTGTTCAATTGTTCAATAAGTCGCTGTGTATGTTTGTTG	Introducing a new right border flanked by 5 T-DNA transfer stimulator sequence repeats as found in pRiA4 (PMID:1515600) flanked by an overdrive sequence, contains half *Pmi*I site at 5' end
P1544	CAACAAACATACACAGCGACTTATTGAACAATTGAACAATTGAACAATTGAACAATTGAAC AAGTTTACCCGCCAATATATCCTGTCAAACACTGAGTTT	Introducing a new right border flanked by 5 T-DNA transfer stimulator sequence repeats as found in pRiA4 (PMID:1515600) flanked by an overdrive sequence, contains half *Pmi*I site at 5' end, reverse
P1677	TGGCGCGCCCAAACCGA	To amplify the *nos* promoter from pX vectors with *Asc*I site
P1678	TGGTACCTCAGATTTCGGTGACGG	To amplify the *pat* (Basta resistence) gene from pX vectors with *Kpn*I site, reverse

P1679	TGGTACCAAGCAGATCGTTCAAACATTTG	To amplify the *nos* terminator from pX vectors (slightly shorter version than in pX) with *Kpn*I site, forward
P1680	TACGCGTCGATCTAGTAACATAGATGACAC	To amplify the *nos* terminator from pX vectors (slightly shorter version than in pX) with *Mlu*I site (*Asc*I compatible ends), reverse
P1997	CGGCCGCCATGGTAA	To amplify the guide array from modified version of pEn-Chimera
P1998	CGGATATAGTTCCTCCTTTCAG	To amplify the guide array from modified version of pEn-Chimera
P2193	GAGGATCATACCTTCTTGATGACC	To amplify a DNA fragment encoding a sgRNA targeting *GSDA.1* (Phvul.007G185600); forward; test-sgRNA1 from the cleavage assay
P2194	AAACGGTCATCAAGAAGGTATGAT	To amplify a DNA fragment encoding a sgRNA targeting *GSDA.1* (Phvul.007G185600); reverse; test-sgRNA1 from the cleavage assay
P2195	GAGGCTGCCTTTGTTAGGAACTTG	To amplify a DNA fragment encoding a sgRNA targeting *GSDA.1* (Phvul.007G185600); forward; test-sgRNA2 from the cleavage assay
P2196	AAACCAAGTTCCTAACAAAGGCAG	To amplify a DNA fragment encoding a sgRNA targeting *GSDA.1* (Phvul.007G185600); reverse; test-sgRNA2 from the cleavage assay
P2197	GAGGGTAACCTCAGCATGCGCAGT	To amplify a DNA fragment encoding a sgRNA targeting *GSDA.1* (Phvul.007G185600); forward; test-sgRNA3 from the cleavage assay
P2198	AAACACTGCGCATGCTGAGGTTAC	To amplify a DNA fragment encoding a sgRNA targeting *GSDA.1* (Phvul.007G185600); reverse; test-sgRNA3 from the cleavage assay
P2199	GAGGTGATCCAACTGCGCATGCTG	To amplify a DNA fragment encoding a sgRNA targeting *GSDA.1* (Phvul.007G185600); forward; test-sgRNA4 from the cleavage assay
P2200	AAACCAGCATGCGCAGTTGGATCA	To amplify a DNA fragment encoding a sgRNA targeting *GSDA.1* (Phvul.007G185600); reverse; test-sgRNA4 from the cleavage assay
P2250	ATGAAGACTTGGAGAAAAATTACGGATATGA	To amplify the parsley ubiquitin promotor and 5'UTR CMPV enhancer of V112 with GGAG - AATG overhangs for level_0 acceptor Pro+5U.
P2251	TAGAAGACAACATTCGCGAATTTGGGCAGAA	To amplify the parsley ubiquitin promotor and 5'UTR CMPV enhancer of V112 with GGAG - AATG overhangs for level_0 acceptor Pro+5U.
P2310	TAGGTCTCCTTCTTGATGACCGTTTTAGAGCTAGAA	To amplify a DNA fragment encoding a sgRNA with tRNA processing system targeting *GSDA.1* (Phvul.007G185600) in *P. vulgaris*
P2311	ATGGTCTCAAGAAGGTATGATTGCACCAGCCGGGAA	To amplify a DNA fragment encoding a sgRNA with tRNA processing system targeting *GSDA.1* (Phvul.007G185600) in *P. vulgaris*
P2312	TAGGTCTCCGTTGCCACAACAGTTTTAGAGCTAGAA	To amplify a DNA fragment encoding a sgRNA with tRNA processing system targeting *GSDA.2* (Phvul.009G220800) in *P. vulgaris*
P2313	ATGGTCTCACAACTGGCAACTTGCACCAGCCGGGAA	To amplify a DNA fragment encoding a sgRNA with tRNA processing system targeting *GSDA.2* (Phvul.009G220800) in *P. vulgaris*
P2314	TAGGTCTCCAGATGCACTGCGGTTTTAGAGCTAGAA	To amplify a DNA fragment encoding a sgRNA with tRNA processing system targeting *GSDA.3* (Phvul.003G124100) in *P. vulgaris*
P2315	ATGGTCTCAATCTGCAATGAATGCACCAGCCGGGAA	To amplify a DNA fragment encoding a sgRNA with tRNA processing system targeting *GSDA.3* (Phvul.003G124100) in *P. vulgaris*
P2325	TAGGTCTCCGCAAACCGGCGAGTTTTAGAGCTAGAA	To amplify a DNA fragment encoding a sgRNA with tRNA processing system targeting *XMPP* (Phvul.007G056000) in *P. vulgaris*
P2329	TAGGTCTCCAGTGTTAGCTGAGTTTTAGAGCTAGAA	To amplify a DNA fragment encoding a sgRNA with tRNA processing system targeting *XDH* (Phvul.005G148000) in *P. vulgaris*
P2331	ATGGTCTCATTGCGAGTCAGTTGCACCAGCCGGGAA	To amplify a DNA fragment encoding a sgRNA with tRNA processing system targeting *XMPP* (Phvul.007G056000) in *P. vulgaris*
P2332	ATGGTCTCACACTCTACGAACTGCACCAGCCGGGAA	To amplify a DNA fragment encoding a sgRNA with tRNA processing system targeting *XDH* (Phvul.005G148000) in *P. vulgaris*
P2498	CTGTGGTATTCCTCTGCGGT	For AFLP of Phvul.007G056000 (*XMPP*)
P2499	GTAAAACGACGGCCAGTAGGGAGAACACGATGTTAACG	For AFLP of Phvul.005G148000 (*XDH*); with M13-tail
P2500	TCCAATTGCACGGTCTACAA	For AFLP of Phvul.005G148000 (*XDH*)
P2501	GTAAAACGACGGCCAGTGAGTCACGGGCTTGTCACTT	For AFLP of Phvul.007G185600 (*GSDA.1*); with M13-tail
P2502	TGTACAGCTGGTATACCAAGAG	For AFLP of Phvul.007G185600 (*GSDA.1*)

P2503	GTAAAACGACGGCCAGTTGAAGATGGTGGTCCCTTTG	For AFLP of Phvul.009G220800 (*GSDA.2*); with M13-tail
P2504	GCATGAATTGCCTAAGAAATCA	For AFLP of Phvul.009G220800 (*GSDA.2*)
P2505	GTAAAACGACGGCCAGTTTTATGGAGCAAAGGCTGAG	For AFLP of Phvul.003G124100 (*GSDA.3*); with M13-tail
P2506	CGAAGGAATCCTGATTAGCC	For AFLP of Phvul.003G124100 (*GSDA.3*)
P2521	GTAAAACGACGGCCAGTGATTTGGACGACACCTTGTACC	For AFLP of Phvul.007G056000 (*XMPP*); with M13-tail
P2561	GGCATCGATAAAATGGGTATTAACTCCCTCGT	To amplify Phaseolus *XMPP* (Phvul.007G056000.1) from cDNA with *Cla*I restriction site for C terminal tagging in V69, forward primer
P2562	GTGCCCGGGAGCTCCAACCAAAGCAA	To amplify Phaseolus *XMPP* (Phvul.007G056000.1) from cDNA with *Xma*I restriction site for C terminal tagging in V69, reverse primer without stop
P2596	ATCCCGGGTCAGGGTTTCATCACCAGCTC	To amplify Phaseolus *NSH1* (Phvul.001G188700) from cDNA with *Xma*I site for N-terminal tagging in V210, reverse primer with stop
P2777	TAGGTCTCCTGTTCACCTGCGGTTTTAGAGCTAGAA	To amplify a DNA fragment encoding a sgRNA with tRNA processing system targeting *NSH1* (Phvul.001G188700) in *P. vulgaris*
P2778	ATGGTCTCATTGTCGGGATTGTGCACCAGCCGGGAA	To amplify a DNA fragment encoding a sgRNA with tRNA processing system targeting *NSH1* (Phvul.001G188700) in *P. vulgaris*
P2779	TAGGTCTCCATTAACTGTATGGTTTTAGAGCTAGAA	To amplify a DNA fragment encoding a sgRNA with tRNA processing system targeting *NSH2* (Phvul.003G000600) in *P. vulgaris*
P2780	ATGGTCTCAATTACAGTCTACTGCACCAGCCGGGAA	To amplify a DNA fragment encoding a sgRNA with tRNA processing system targeting *NSH2* (Phvul.003G000600) in *P. vulgaris*
P2789	ACCATGGCCTCGCAGGTGAAC	To amplify *P. vulgaris NSH1* (Phvul.001G188700) from cDNA with *Nco*I site, forward
P2790	ACCATGGCAGCCGATACCGAAC	To amplify *P. vulgaris NSH2* (Phvul.003G000600) from cDNA with *Nco*I site, forward
P2791	TCCCGGGTCAAGAGTCCAAAAGGCGATC	To amplify *P. vulgaris NSH2* (Phvul.003G000600) from cDNA with *Xma*I site, reverse
P2792	ACCATGGCAGCCGATACCGAACCAAAGAAGATCATAATTGATACCGACCCTGGCATTGATG ATGCTATGGCAATATTTGTTGC	To remove *Cla*I and *Nco*I sites from *NSH2* (Phvul.003G000600) from cDNA by PCR
P3085	TGGCGCGCCTCAATTAATGGGTAAGGTGATA	To amplify a 3 kb version of the *P. vulgaris XDH* promoter, Phvul.005G148000, forward with *Asc*I site
P3088	TGAATTCGGAAACGAACGGAACGG	To amplify a 3 kb version of the *P. vulgaris XDH* promoter, Phvul.005G148000, reverse with *EcoR*I site
NSH1 DNA	CATATGCAAATTCTATAGGGACTGGCATGCAAAATCTGATGGTGTTCATGGAATTTTCCTC CACGATCCCGTCAGTTTTGTGGCCCTTGTGCGTCCAGATCTTTTTACATACCAGAAAGGGG TTGTGAGGGTAGAGACACAAGGCATCTGTGTTGGGCATACACTTCTGGATCAAGGATTGAA AAATTGGAATATGAGCAACCCTTGGACAGGTTATTCTCCTGTTTCAGTTGTTTGGACCGTG GATGTCGAGGGAGTTGTTGATTATATCAAGGAGCTGGTGATGAAACCCTGACCCGGG	Synthetic *NSH1* (Phvul.001G188700) DNA fragment to remove two internal *Nco*I sites

Supplementary Table 2 LC-MS parameters.

MS source parameter	Positive ion mode	Negative ion mode
Ion source	AJS ESI	AJS ESI
Gas temperature	300°C	350°C
Gas flow	12 l min^{-1}	12 l min^{-1}
Nebulizer	30 psi	40 psi
Sheath gas heater	300°C	350°C
Sheath gas flow	11 l min^{-1}	12 l min^{-1}
Capillary	4,000 V	2,500 V

Analyte	Ion mode	Retention time (min)	Precursor ion (m/z)	Product ions (m/z)[1]	Fragmentor (V)	Collision energy (V)	Qualifier ratio
XMP	Pos.	1	365.0	212.9	84	3	
				153.0	84	15	58.6-85.9
Xanthosine	Pos.	1.8	285.1	153.0	60	5	
				135.9	60	35	13.6-23.3
Xanthine	Pos.	2.4	153.1	109.0	100	15	
				55.2	100	33	47.9-75.1
Guanosine	Pos.	5.4	284.1	152.0	90	10	
				135.0	90	45	26.0-39.5
Guanine	Pos.	2.2	152.1	135.0	118	18	
				110.0	118	22	43.9-66.5
Inosine	Pos.	6	269.1	136.9	55	14	
				118.9	55	40	7.9-12.1
Hypoxanthine	Pos.	2.5	137.1	118.9	117	22	
				110.0	117	22	67.6-95.3
Allantoin	Neg.	17	157.0	113.9	96	15	
				96.9	96	15	96.6-144.0
Allantoate	Pos.	0.9	177.1	117.0	60	25	
				74.0	60	25	21.9-36.1

[1]The first listed product ion was used for quantification.
