# Supplementary material for: Enzymes and cellular interplay required for flux of fixed nitrogen to ureides in bean nodules
Source: Nat Commun. 2022 Sep 10;13:5331. doi: 10.1038/s41467-022-33005-5 (PMC9464200; doi:10.1038/s41467-022-33005-5)
Supplement: Supplementary file 3 — Reporting Summary [file 41467_2022_33005_MOESM3_ESM.pdf]

nature portfolio

Corresponding author(s): Claus-Peter Witte

Last updated by author(s): Aug 22, 2022

Reporting Summary

Nature Portfolio wishes to improve the reproducibility of the work that we publish. This form provides structure for consistency and transparency in reporting. For further information on Nature Portfolio policies, see our Editorial Policies and the Editorial Policy Checklist.

Statistics

For all statistical analyses, confirm that the following items are present in the figure legend, table legend, main text, or Methods section.

n/a	Confirmed	
☐	☒	The exact sample size (*n*) for each experimental group/condition, given as a discrete number and unit of measurement
☐	☒	A statement on whether measurements were taken from distinct samples or whether the same sample was measured repeatedly
☐	☒	The statistical test(s) used AND whether they are one- or two-sided<br>*Only common tests should be described solely by name; describe more complex techniques in the Methods section.*
☒	☐	A description of all covariates tested
☐	☒	A description of any assumptions or corrections, such as tests of normality and adjustment for multiple comparisons
☐	☒	A full description of the statistical parameters including central tendency (e.g. means) or other basic estimates (e.g. regression coefficient) AND variation (e.g. standard deviation) or associated estimates of uncertainty (e.g. confidence intervals)
☐	☒	For null hypothesis testing, the test statistic (e.g. *F*, *t*, *r*) with confidence intervals, effect sizes, degrees of freedom and *P* value noted<br>*Give P values as exact values whenever suitable.*
☒	☐	For Bayesian analysis, information on the choice of priors and Markov chain Monte Carlo settings
☒	☐	For hierarchical and complex designs, identification of the appropriate level for tests and full reporting of outcomes
☒	☐	Estimates of effect sizes (e.g. Cohen's *d*, Pearson's *r*), indicating how they were calculated

Our web collection on statistics for biologists contains articles on many of the points above.

Software and code

Policy information about availability of computer code

Data collection	Leica Application Suite X (Ver. 3.7.2.22383), MassHunter Workstation Software LC/MS Data Acquisition for 6400 Series Triple Quadrupole (Ver. B.07.00), UV Probe (Ver. 2.51), NIS-Elements Viewer (Ver. 4.20), GeneMapperID (Ver. 3.2), CLC Genomics Workbench (Ver. 7.5.5)
Data analysis	Excel 2016, Muscle (at the website of the European Bioinformatics Institute), GraphPad Prism 4, R software (Ver. 1.2.5042), MassHunter Workstation Software Quantitative Analysis (Ver. B.09.00), pyBoxshade (Ver. 1.2)

For manuscripts utilizing custom algorithms or software that are central to the research but not yet described in published literature, software must be made available to editors and reviewers. We strongly encourage code deposition in a community repository (e.g. GitHub). See the Nature Portfolio guidelines for submitting code & software for further information.

Data

Policy information about availability of data

All manuscripts must include a data availability statement. This statement should provide the following information, where applicable:

- Accession codes, unique identifiers, or web links for publicly available datasets
- A description of any restrictions on data availability
- For clinical datasets or third party data, please ensure that the statement adheres to our policy

RNA-Seq data were obtained from the NCBI short Read Archive (SRA, accession PRJNA322355 for M. truncatula; PRJDB2819 for L. japonicus; PRJNA322335 for P.vulgaris; PRJNA79597 and PRJNA208048 for G. max). Sequence data of genes mentioned in this article can be found with the locus identifiers for XMPP (Phvul.007G056000), GSDA.1 (Phvul.007G185600), GSDA.2 (Phvul.009G220800), GSDA.3 (Phvul.003G124100), NSH1 (Phvul.001G188700), NSH2 (Phvul.003G000600), XDH (Phvul.005G148000), UOX (Phvul.007G234300) and ALN (Phvul.006G186700). Because the sequences were obtained from the bean

cultivar Negro Jamapa, there are a few sequence differences to the published genome. The exact sequences that were used in this study can be found in the source data file.

Field-specific reporting

Please select the one below that is the best fit for your research. If you are not sure, read the appropriate sections before making your selection.

☒ Life sciences ☐ Behavioural & social sciences ☐ Ecological, evolutionary & environmental sciences

For a reference copy of the document with all sections, see nature.com/documents/nr-reporting-summary-flat.pdf

Life sciences study design

All studies must disclose on these points even when the disclosure is negative.

Sample size	For metabolite analyses of Phaseolus nodules, sample sizes varied from n=3 to n=5 biological replicates. A replicate was a nodule pool from a single transgenic root. Because harvesting and genotyping of the possible edited nodule mutant pools was laborious, the number of samples was highly dependent on the resulting number of identified null-mutant nodule pools. For characterization of XDH CRISPR-transformed Soybean nodules, sample sizes were n = 22 for category I, n = 18 for category II, n = 5 for category III and n = 29 for control. However, nodules were not tested for mutations. A replicate was a nodule pool from a single transgenic root.
Data exclusions	In the metabolite analysis measurements failing the pre-established quality criteria for retention time, qualifier to quantifier ratio or with a signal to noise ratio below 10 were called “not detected” (see Supplementary Table 2).
Replication	In vitro enzyme activities and (sub)cellular localization in nodules were reproduced at least twice. The Cas9 in vitro cleavage assay for sgRNAs targeting GSDA.1 was reproduced twice. For enzyme activities, three technical replicates were made.
Randomization	Plants used for metabolite analysis were grown and harvested randomized.
Blinding	After plant and nodule harvest, samples were analyzed in randomized and blind fashion.

Reporting for specific materials, systems and methods

We require information from authors about some types of materials, experimental systems and methods used in many studies. Here, indicate whether each material, system or method listed is relevant to your study. If you are not sure if a list item applies to your research, read the appropriate section before selecting a response.

Materials & experimental systems

n/a	Involved in the study	
☐	☒	Antibodies
☒	☐	Eukaryotic cell lines
☒	☐	Palaeontology and archaeology
☒	☐	Animals and other organisms
☒	☐	Human research participants
☒	☐	Clinical data
☒	☐	Dual use research of concern

Methods

n/a	Involved in the study	
☒	☐	ChIP-seq
☒	☐	Flow cytometry
☒	☐	MRI-based neuroimaging

Antibodies

Antibodies used	Strep-Tactin alkaline phosphatase conjugate antibody (IBA Lifesciences, 2-1503-001, 1503-0014); anti c-myc antibody (Roche, 11667149001, 28402100); anti-mouse IgG alkaline phosphatase conjugate antibody (Sigma-Aldrich, A3562, SLCD1906)
Validation	According to the manufacturers' website, the Strep-Tactin AP antibody is suitable for direct detection for N-, C- or internally tagged Strep-tag fusion proteins transferred to a blotting membrane. According to the manufacturers' website, the anti c-myc antibody is tested for functionality and purity relative to a reference standard to confirm the quality of each new reagent lot. The certificate of analysis of the anti-mouse antibody can be obtained from the manufacturers' website.
